# Evaluation of the Anti-Inflammatory Effect of Locally Delivered Vitamin C in the Treatment of Persistent Gingival Inflammation: Clinical and Histopathological Study

**DOI:** 10.1155/2016/2978741

**Published:** 2016-12-05

**Authors:** Nermin M. Yussif, Manar A. Abdul Aziz, Ahmed R. Abdel Rahman

**Affiliations:** ^1^National Institute of Laser Enhanced Sciences, Cairo University, Giza, Egypt; ^2^Oral Pathology Department, Faculty of Oral & Dental Medicine, Cairo University, Giza, Egypt; ^3^Oral Diagnosis, Medicine & Periodontology Department, Faculty of Oral & Dental Medicine, Cairo University, Giza, Egypt

## Abstract

*Objective*. The purpose of this study is to investigate the role and efficiency of the locally injected vitamin C in the treatment of persistent gingival inflammation.* Design*. Twenty adult patients with persistent chronic gingival inflammation were included in this study. The same dose of sterile vitamin C was injected in gingival tissues after the completion of phase I therapy. Gingival biopsies were taken after total resolution of inflammation. The specimens were examined histologically, using H&E stain.* Results*. Clinical evaluation revealed great improvement of the injected sites with recall visits. Histopathological results revealed marked decrease in inflammatory cells and epithelial thickness and a higher number of newly formed subbasal capillaries.* Conclusions*. Vitamin C is an effective adjunctive treatment in reducing various degrees of chronic gingival inflammation.

## 1. Introduction

Plaque induced gingivitis is the most common oral disease affecting the gingival tissues [1]. Infected dental plaque biofilm and the impaired host response remain the main causes of a wide range of gingival diseases. The ability of the patient to adhere to a proper oral care plan is also a critical factor that controls the tissue reaction and disease progression [2].

The clinical manifestations of gingival inflammation differ according to the severity, distribution, and the response to treatment ranging between being localized and highly responsive to treatment to long standing lesions with persistent redness and massive bleeding on probing with variable degrees of swelling [3]. The efficacy of treatment and the subsequent tissue response vary according to the etiology and idiopathic, local, or systemic cause [2].

Under healthy conditions, the neutrophils pass from the gingival connective tissue through the junctional epithelium into the gingival sulcus, providing a dynamic balance between continuously invading microorganisms and the immune response [4].

The antioxidants, either enzymatic or nonenzymatic, play a crucial role in the tissue equilibrium via neutralizing such harmful agents (ROS) [5, 6].

Under certain inflammatory conditions, the total amount of antioxidants decreases to its lowest levels, which necessities their supplementation by external source [7, 8].

**Figure 1:**
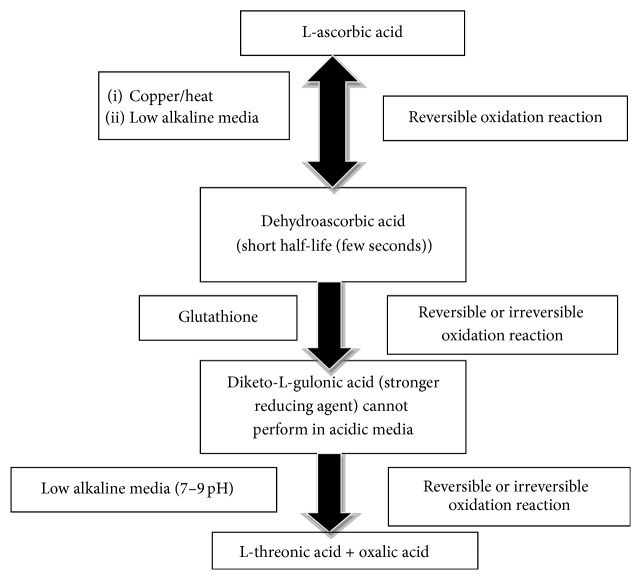
Scheme of the biological function of ascorbic acid as an antioxidant.

One of the most important antioxidant agents is vitamin C (vit-C) which has an important role in different body functions [7, 8]. Despite its mild acidity (pH 4.2), it requires an alkaline medium to perform its function as a reducing agent [9]. To be activated, many changes in its structure occur in three successive cycles of oxidation (Figure 1).

Vitamin C, being a strong reducing agent, can regulate the resolution of the inflammatory process and stimulate the tissue repair. It modulates the release of catabolic inflammatory cytokines [10–12], chemotaxis of the immune cells, and activation of the phagocytosis [4]. The neutrophils and lymphocytes containing vitamin C show lower apoptotic potentiality and higher proliferation rate. It also increases the production of antibodies [4].

Furthermore, it enhances the synthesis of collagen type I, reduced by inflammatory process, keeps the balance between collagen I and collagen III, and modifies the rate of fibroblast proliferation. It also reduces the potentiality of scaring via inhibiting cross-linking of collagen fibers and fibrosis. Vitamin C is also essential for new blood vessel formation (angiogenesis). It acts as a cofactor in hydroxyproline synthesis to produce collagen type IV and improves endothelial cell vitality and function [10–13].

The aim of the present study is to investigate the exact role and efficiency of the locally injected vitamin C in the treatment of persistent gingival inflammation after single or multiple dosing based on clinical and histopathological examination.

## 2. Methodology

### 2.1. Ethical Aspects

Each patient signed an informed detailed consent form before participation, explaining the benefits, steps, and side effects of the treatment protocol.

### 2.2. Study Design

The study was designed as a single arm, unblinded, and unrandomized trial as the trial did not depend on patient selection but it was rather based on an detection of a chronic persistent phenomenon. Only patients with persistent inflammation were included.

### 2.3. Screening Procedures

**Figure 2:**
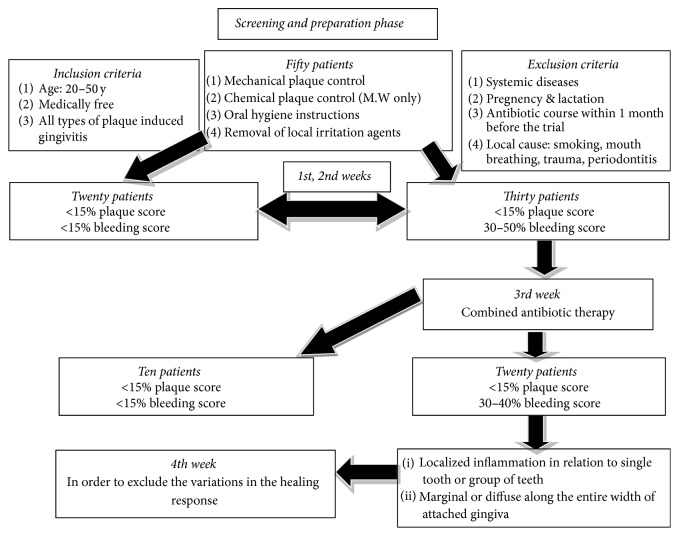
Scheme represents the procedure steps.

Patients were recruited among those diagnosed with plaque induced gingivitis in the postgraduate periodontology clinic at Cairo University between the years 2013 and 2016. An initial evaluation, including medical and dental history, clinical examination, and radiographic examination, was conducted by NMY and ARA to determine patient eligibility for the study. Fifty patients who met the following inclusion criteria were examined: (1) aged between 20 and 50 y, (2) medically free, and (3) generalized plaque induced gingivitis. All the reasons that could provoke an inflammatory reaction were excluded: (1) systemic diseases, (2) pregnant and lactating mothers, (3) treatment with antibiotic medication within 1 month before the trial, and (4) local causes (smoking, mouth breathing, local trauma, and periodontitis) (Figure 2).

### 2.4. Patient Preparation

Each patient underwent full-mouth sessions of supragingival debridement using ultrasonic and hand instrumentation and received personalized oral hygiene instructions. In addition, chlorhexidine 0.12% mouth rinsing was recommended twice daily. All patients were placed on 2-week maintenance recall appointments. About twenty patients achieved a minimal residual inflammation (>15% of plaque and bleeding scores according to O'Leary's scoring system) and optimal soft tissue conditions during two weeks. Combination antibiotic therapy (Amoxicillin 500 mg and Metronidazole 500 mg/3 times per day for 7 days) was administrated in order to control the amount of the condition. After 7 days of the complexion of the antibiotic course, reevaluation of the remaining 30 patients was undergone. At the end of the maintenance period (4 weeks), only the refractory cases were included in the study. The included patients were 20 who suffered from persistent gingival inflammation in the esthetic anterior region (Figure 2).

### 2.5. Procedure

The same examiner (NMY) performed all injection procedures. The site of interest was anesthetized using lidocaine-epinephrine 1 : 100,000. Intraepidermal injection (mesotherapy approach) of 1–1.5 mL (200–300 mg concentration) of L-ascorbic acid was locally introduced in relation to the keratinized gingival tissues with prevalent extension to the whole target region, respectively, using insulin syringes. The recommended dosage is equivalent to the region extending between bilateral canines. The current dose is equivalent to the treated region (distance extends between six maxillary anteriors) [14]. During the first injection visit, it is recommended to use half the permissible dose only. The same dose was repeated once per week until inflammation subsided.

### 2.6. Postoperative Instructions

At the end of each session, patients were prescribed a rescue analgesic (Ibuprofen 200 mg) to be used as needed. The patients were asked to abstain from mechanical oral hygiene procedures in relation to the target region for the day of procedure only.

### 2.7. Clinical Parameters

Immediately before injection procedure, the following clinical measurements were performed by the same examiners (NMY and ARA): plaque and bleeding score using O'Leary's scoring system [15] and local bleeding score using Sulcus bleeding index (SBI) [16] as a more specific index:

Score 0: healthy looking and no bleeding on probing
Score 1: healthy looking and bleeding on probing
Score 2: bleeding on probing, change in color, and no edema
Score 3: bleeding on probing, change in color, and slight edema
Score 4: bleeding on probing, change in color, and obvious edema
Score 5: spontaneous bleeding, change in color, and marked edema

The latter index examined color, swelling, and bleeding tendency. At the completion of the injection visits, the same indices were used to detect changes. Digital photographs were taken preoperatively and 2 weeks postoperatively. Patient satisfaction was performed by using a 5- graded self-assessment analysis: excellent (4), improved over 75%; good (3), improved 50–75%; moderate (2), improved 25–50%; fair (1), improved less than 25%; no change or worse (0), not improved or darkened [17].

### 2.8. Histopathological Examination

Preoperative and postoperative (after 1 week of last injection) gingival biopsies were excised, immediately fixed in 10% neutral buffered formalin, and then processed in the routine way for preparing a paraffin block. Tissue sections were cut and stained with hematoxylin & eosin (H&E) for histopathological examination. Finally, epidermal and dermal changes before and after ascorbic acid application were assessed using computer image analyzer software Leica QWin 515 system (England).

## 3. Results

### 3.1. Study Population

Twenty patients were included in the study. The experimental period was between September 2013 and April 2016 (last follow-up visit). Twenty sites of localized chronic gingival inflammation were included with no definite cause ranging between marginal gingivitis and diffuse gingivitis. None of the patients was excluded from the study.

### 3.2. Clinical Scores

**Figure 3:**
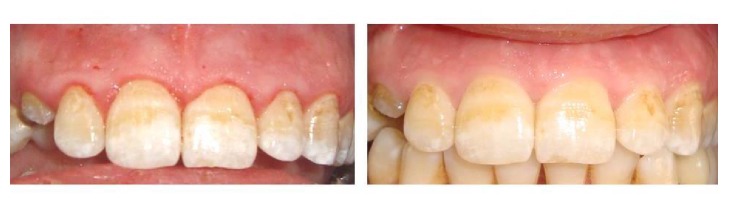
*Preoperative photo*: this patient was referred from the oral surgery department for hygiene reinforcement prior to immediate implant in relation to the upper right canine.* Postoperative photo* revealed total improvement of the gingival inflammation after 2 vitamin C injections.

**Figure 4:**
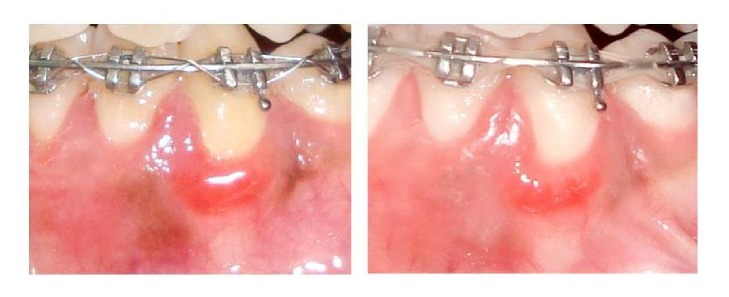
*Preoperative photo*: this patient was referred from the orthodontic department suffering from severe bleeding gums.* Postoperative photo* revealed partial improvement of the gingival inflammation after 2 vitamin C injections. The patient needed further injection for total improvement.

The age of the enrolled patients ranged between 20 and 50 years. Most of the involved patients were females (90%). Improvement was reported in all cases after maximum 2 injections except 2 cases that needed one more injection. Measurement of the SBI was done following phase I therapy and after the completion of the treatment. All the treated patients respond positively to the applied treatment. Preoperatively, it was noted that 12 (60%) out of 20 patients scored 3 and 8 patients (40%) scored 4 on SBI index. Postoperatively, the range changed into 20 patients (100%) scoring zero with variable degree of response to the applied treatment. Seven patients (35%) out of twenty treated patients were totally free of inflammation with zero SBI score after 1 injection visit. Eleven (55%) patients showed the same results after 2 injection visits. Only 2 patients (10%) out of twenty needed further injection to reach the zero score. All the enrolled patients showed great satisfaction about the treatment results, even the patients who were not fully treated. All patients (100%) showed great satisfaction with the results (score 4) (Figures 3 and 4).

### 3.3. Microscopic Examination of H&E Stained Sections

#### 3.3.1. Preoperative Microscopic Examination

**Figure 5:**
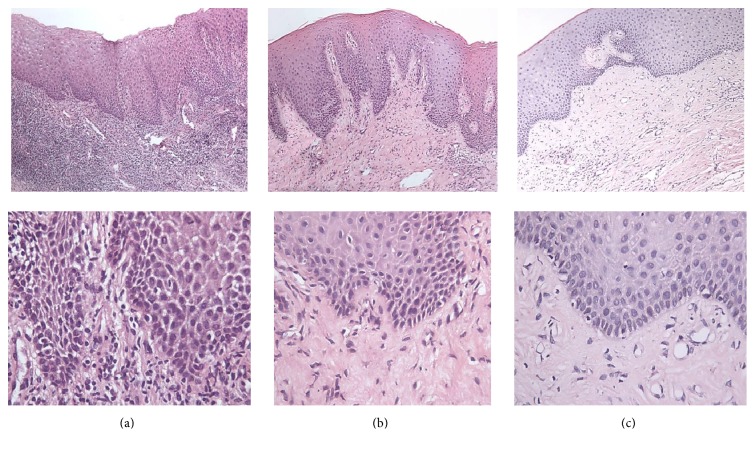
(a) Photomicrograph of chronically inflamed gingival tissue (preoperative) showing hyperplastic epithelium with long and broad rete ridges and intense inflammatory cell infiltrate in superficial and deep CT (×100). Intraepithelial edema and intense inflammatory cell infiltrate formed of acute and chronic inflammatory cells (×400). (b) Photomicrograph of the same case after single injection of vit-C showing hyperplastic epithelium with slightly narrower rete ridges, numerous minute blood capillaries, collagen fibers formation, and mild inflammatory cell infiltrate (×100). Reduced intercellular edema with fewer chronic inflammatory cells and collagen fibers formation were detected (×400). (c) Photomicrograph of the same case after two injections (in two sessions) of vit-C showing marked reduction in epithelial thickness, superficial minute, and deep larger blood capillaries, collagen fiber bundles formation, and mild inflammatory cell infiltrate (×100). Marked reduction of vacuolized epithelial cells, reduced intercellular edema, well-demarcated basement membrane, increased number of blood capillaries, and almost absence of inflammatory cells (×400).

The whole tissue section revealed severe chronic inflammatory process. The surface epithelium of parakeratinized stratified squamous type was hyperplastic with broad and elongated rete pegs, forming numerous epithelial arcades. Obvious intracellular edema and extracellular edema of epithelial cells disturbing the intercellular desmosomal junctions were seen. Basal cells were crowded in some areas. An intense acute and chronic inflammatory cells infiltrate was observed dispersed in the whole thickness of connective tissue along with coarse blood capillaries (Table 1). Basement membrane was masked in some area by inflammatory cells (Figure 5).

#### 3.3.2. Postoperative Microscopic Examination

*After 1 Week of Single Injection*. Microscopic examination revealed lower grade of inflammation. On the contrary to the preoperative sections, a less hyperplastic surface epithelium with shorter and narrower rete pegs was detected. The intracellular edema and extracellular edema decreased with almost intact desmosomal junctions. Few epithelial arcades were also detected. The basal cells became more regular and well aligned. In some areas, mild signs of inflammation were detected. The connective tissue was formed of proliferating fibroblasts, collagen fiber formation, and few aggregates of chronic inflammatory cells. The characteristic phenomena were the presence of vacuolated epithelial cells especially in basal cell layer (at the site of injection) and the appearance of minute capillaries in subepithelial CT areas (Figure 5).

*After 1 Week of Twice Injections*. Examination of the postoperative sections revealed a great improvement. Marked well defined parakeratinized layer was detected. The epithelial hyperplasia disappeared with intact intercellular desmosomal junctions. Obvious reduction in the epithelial thickness was detected with minimal epithelial rete pegs and epithelial arcades. Higher incidence of vacuolated cells was detected rather than the two injection sections. The basement membrane was clearly seen. Well-formed collagen fiber bundles with numerous and widely distributed blood vessels were detected in the connective tissue. The same phenomenon of subepithelial minute capillaries was clearly found. Fewer chronic inflammatory cells were detected but restricted to the deep connective tissue (Figure 5).

## 4. Discussion

Due to diversity of the gingival diseases, treatment plan should be variably designated based on the associated causative factors. However, one of the embarrassing problems is the presence of chronic or resistant inflammatory conditions with no definite aetiopathogenesis. Such conditions persist even with removal of all apparent causes and oral hygiene reinforcement. They are usually characterized by persistent redness and bleeding on probing with different degrees of tissue edema which may be localized or diffused [2].

In these cases, massive antibiotic course or corticosteroids regimens followed by surgical intervention if needed were the conventionally and commonly used protocols. Despite the incredible results accompanying corticosteroids, they are usually avoided due to their adverse effects.

Therefore, there is a need for other safe nonsurgical therapeutic agents in order to control such conditions or, at least, improve the tissue healing and reduce inflammatory manifestations prior to the surgical removal of remaining pseudopocketing. Dermal mesotherapeutic techniques using antioxidants such as vit-C showed promising results in the treatment of similar localized inflammatory conditions [18].

The aim of the current study is the regulation of the overresponse of the inflammatory process, promoting healing, increasing the collagen content of the affected tissues, and improving the gingival circulation.

In the current study, twenty patients were enrolled and evaluated at the baseline (after 4 weeks following phase I therapy) and postoperatively in the recall visit (after 1 week after the last intraepidermal vitamin C injection). The long follow-up period preceding the therapy was essential in order to ensure that gingival inflammation is resistant to the conventional therapy.

During inflammation, it was found that the tissue antioxidant level (vit-C, vit-E, etc.) decreases rapidly, indicating the need of its supplementation. On the other hand, the free radicals production increases at the site of inflammation [19, 20]. Extra doses of antioxidants, especially vitamin C, are essential. In localized inflammatory conditions, the administration of the needed higher doses (higher than 500 mg) cannot be absorbed by the gastrointestinal tract which easily excreted through urine. Moreover, in order to reach this dose at the site of inflammation, administration of very high systemic doses is needed, which could be harmful to the patient. The local injection provides the needed dose efficiently [21].

Among Egyptians, the incidence of vitamin C deficiency is nearly neglected due to its availability in a wide range of fruits and vegetables. Due to the impaired financial status of our patients and the absence of the deficiency conditions, the used technique is much cheaper than providing a long term special food protocol or oral supplementations in order to improve their health status.

The local injection of vit-C was preferred rather than the usage of topical vitamin C gel or dentifrice that was previously used by Daniels and Jefferies [22] and Shimabukuro et al. [23]. Vitamin C is a water-soluble agent that has superficial penetrating effect. Many studies used different forms of lipid soluble topical vitamin C in order to overcome its limited absorption. These studies met several limitations such as very long term improvement time (12 weeks), easily dislodged from the oral tissues, limited absorption, instability when exposed to solutions, air, heat, or light, and localized enamel erosions [9, 24, 25].

Vitamin C was injected at the subepithelial level. Total numerical evaluation of the gingival inflammation (using SBI) was determined, resulting in an average value per each patient. This analysis indicates 100% reduction in gingival inflammation from the average baseline value over the 7–21 days. The reported percent indicated significant reduction in the gingival inflammation. Intraepidermal vitamin C injection could be either single or double or triple with one week apart according to the severity of the condition. More cautious handling of the inflamed tissues during the first visit is quite important in order to avoid tearing or necrosis, especially in thin biotype tissues. Improvement of the tissue color and form was clearly observed in the recall visits after 2 months.

Once inflammation subsided, representative tissue biopsies have been obtained 7 days following the last injection from the patients indicated for gingivoplasty or gingivectomy. Clinically significant reduction of the pseudogingival enlargement was associated with return of the basal keratinocytes to their normal proliferative pattern which is the main role of vitamin C [26, 27]. The anti-inflammatory action of vit-C was also evident and was detected in our specimens as a reduced intraepithelial edema and inflammatory cells.

Other characteristic changes which occurred after vit-C administration were in accordance with Nusgens et al. [28]. An increased number of fibroblasts were clearly detected forming more collagen fibers that showed more maturation and bundles formation following second injection. Numerous newly formed small blood capillaries were also detected. These features are consistent with its known essential role in the formation of new connective tissue in a healing wound. This is because it acts as a cofactor for enzymes critical in collagen formation, the main component of the connective tissue that forms the framework around which the new tissue is rebuilt. Collagen also represents an essential component in the wall of blood vessels. This is why, despite the increase in number of blood vessels, redness and bleeding tendency markedly decreased clinically. These vessels provide more nutritional and oxygen support to chronically irritated and continuously damaged mucosal areas, improving their healing.

Our results were in accordance with Boyce et al. They detected that vitamin C promotes the development of an organization of the basement membrane and also restores the epidermal barrier within 2-3 weeks. Furthermore, it promotes the wound closure and reduces its contraction which limits the incidence of scare formation [27].

Histologically, a characteristic cellular vacuolization was observed in all groups. In the preoperative specimens, vacuolated cells appeared as clear cells with small pyknotic dark nucleus representing signs of degeneration. They were found in clusters widely distributed throughout all epithelial layers. In contrast, vit-C associated vacuolated cells were individually distributed along the epithelial layers with greatest aggregations in basal cells. The nuclei were rounded or kidney shaped with nearly normal stains. A similar vacuolization was found in immediate and 15-minute biopsies excised after intradermal injection of local anesthesia described by Kimura et al. [29]. They attributed this phenomenon to the injection procedure itself rather than the used solution spatially with the presence of vacuolization in biopsies. However, the presence of these cells in after-week biopsies may be due to the ability of vitamin C to increase the cell resistance to death. Furthermore, the basal localization of vacuolated cells, adjacent to injection, may indicate proper infusibility of vit-C.

All these positive clinical changes were met by further patients' cooperation and resulted in their end-treatment satisfaction.

These data suggest a significant enhancement of the gingival health by the usage of the antioxidant approach. Finally, we recommend the usage of the intraepidermal vitamin C injection as an adjunctive approach for the conventional nonsurgical treatment modality. Further studies with long term follow-up are recommended.

## Figures and Tables

**Table 1:** Microscopic features of preoperative and postoperative specimens are summarized.

Tissue components	Preoperative	1st postoperative	Postoperative
*Surface epithelium*			
Epithelial thickness	Hyperplastic	Less hyperplastic	Almost normal
Intercellular edema	Severe	Markedly reduced	Absent
Epithelial cell vacuolization	Degenerated cells	Present	Present
Basal cell alignment	Crowded	Well aligned	Well aligned
Basement membrane	Masked in some areas	Detected	Well formed
Inflammatory infiltrate	Present in some area	Absent	Absent
*Connective tissue*			
Acute inflammatory cells	Present in subepithelial CT	Absent	Absent
Chronic inflammatory cells	Present in subepithelial and deep CT	Present in deep CT	Present in deep CT
Collagen fibers	Few	More	Forming bundles
Blood vessels	Few and large	More	More
Subepithelial minute blood capillaries	Absent	Present	Numerous
